# LPS alters pattern of sickness behavior but does not affect glutathione level in aged male rats

**DOI:** 10.1007/s10522-016-9636-x

**Published:** 2016-02-01

**Authors:** Sylwia Wrotek, Tomasz Jędrzejewski, Anna Nowakowska, Wiesław Kozak

**Affiliations:** Department of Immunology, Nicolaus Copernicus University, Lwowska 1, 87-100 Torun, Poland; Department of Animal Physiology, Nicolaus Copernicus University, Lwowska 1, 87-100 Torun, Poland

**Keywords:** Aging, Sickness behavior, Inflammation, Glutathione, IL-6

## Abstract

Behavioral symptoms of sickness, such as fever and motor activity are a coordinated set of changes that develop during infection. The aim of study was to compare the sickness behaviour (SB) in healthy old and young rats treated with pyrogenic dose of endotoxin and to check their glutathione level. Before experimentation male Wistar rats were selected according to standard body mass, motor activity, and white blood cells count. Intraperitoneal injection of lipopolysaccharide (LPS) from *E. coli* was used to provoke SB. The level of liver glutathione, interleukin (IL) -6, deep body temperature (Tb) and motor activity were measured. Glutathione level in old and young rats did not differ significantly. In both young and old rats LPS administration provoked fever (the mean value of Tb was 38.06 ± 0.01 °C in old rats, and 38.19 ± 0.06 °C in young rats). LPS injection affected night-time activity in both groups (12 h averages were 1.56 ± 0.40 counts in old LPS-treated rats vs 2.74 ± 0.53 counts in not-treated old rats and 3.44 ± 0.60 counts for young LPS-treated vs 4.28 ± 0.57 counts for young not-treated rats). The injection of LPS provoked an elevation of plasma IL-6 concentration (from values below the lowest detectable standard in not-treated groups of animals to 6322.82 ± 537.00 pg/mL in old LPS-treated rats and 7415.62 ± 451.88 pg/mL in young LPS-treated rats). Based on these data, we conclude that good health of aged rats prevents decrease in the glutathione level. Old rats are still able to develop SB in response to pyrogenic dose of LPS, although its components have changed pattern compared to young animals.

## Introduction

Sickness behavior (SB) develops in ill individuals during the course of an infection and trauma. It is manifested with lethargy, depression, decreased motor activity, anxiety, loss of appetite, sleepiness, hyperalgesia, reduction in grooming, fever or anapyrexia (Kelley et al. 2003; Hart 1991; Kozak 1997; Braun and Marks 2010; Mullington et al. 2000; Maier et al. 1993; Dantzer and Kelley 2007). Sickness behavior reorganizes the organism’s priorities to cope with infectious pathogens (Johnson 2002; Kluger et al. 1996), e.g. it has been found that fever creates optimal conditions for the immune processes such as proliferation and differentiation of T cells, secretion of interferons, antibodies, and neutrophil migration (Roberts 1991).

It is commonly accepted that older individuals usually have increased susceptibility to diseases and increased morbidity and mortality due to infections (Singh and Newman 2011; Freund et al. 2010). On the other hand, there are also centenarians who have grown old in relatively good health (Engberg et al. 2009) and who may effectively recover from infections.

The nature of mechanisms that cause the aging remains one of the most poorly understood biological phenomenon. There are many theories which are trying to explain the aging process, each from its own perspective, and none of the theories can explain all details of it. The main molecular characteristic of aging is the progressive accumulation of damages in macromolecules. There are three major sources of damage within a cell: nutritional glucose, spontaneous errors in biochemical processes and reactive oxygen species (Rattan 2006).

Harman first proposed that normal aging is a result of an action of free radicals which cause random deleterious damage of tissues (Harman 1956) and subsequently he extended the idea to implicate mitochondrial production of reactive oxygen (Harman 1972). According to this theory, mitochondria are the major source of toxic oxidants, and enhanced and unopposed metabolism-driven oxidative stress has a major role in diverse chronic age-related diseases. Halliwell and Gutteridge (2007) later suggested renaming this free radical theory of aging to “oxidative damage theory of aging”, since aging is caused not only by free radicals, but also by other chemically reactive molecules such as reactive oxygen (ROS) and nitrogen (NOS) species. Oxidative stress hypothesis holds that the rate of aging is a function of imbalance between those chemically reactive molecules and the antioxidant defences. Consequently, we can observe structural damage which accumulates with time causing corresponding losses in function (Sohal and Orr 2012).

In order to prevent oxidative damage, all living organisms developed a variety of antioxidants (Valko et al. 2007). Among them, glutathione is the major intracellular redox buffer in various cell types. Glutathione is also a powerful stimulator of immune function: T lymphocyte proliferation (Sido et al. 2000; Hadzic et al. 2005), phagocytic activity of polymorphonuclear neutrophils (Ghezzi 2011), dendritic cell functions (Kuppner et al. 2003). It is also an important agent for the first step of the adaptive immunity, consisting of the antigen presentation by antigen presenting cells (Peterson et al. 1998). Accumulating evidence suggests that glutathione affects the secretion of various cytokines, including those associated with fever and inflammation (Haddad and Land 2002). It is known that in the performance of their function, the immune cells may exhaust their antioxidant reserves, which is easy to observe in organism that suffer from chronic inflammation (Townsed et al. 2003). In our previous paper, we have found that rats with low glutathione level react on the endotoxin with significantly attenuated fever (Wrotek et al. 2015).

In a current study we strictly selected healthy, old rats. We excluded rats with loss of body mass, with altered white blood cells level, and apathetic animals. This procedure allowed us to minimize the participation of animals with ongoing, subclinical inflammation that according to current knowledge, may affect the glutathione level and fever. The aim of our study was to check glutathione level in healthy old rats and to investigate their SB provoked by pyrogenic dose of endotoxin.

## Materials and methods

Eight weeks old rats were obtained from Experimental and Clinical Medical Institute Warsaw (Poland). Due to strong influences of reproductive cycles on physiological responses of female rats all experiments were performed on male subjects only. They were housed in plastic cages and placed in a temperature/humidity/light-controlled chamber set at 22 ± 1 °C, 12:12 h light:dark cycle, with light on at 7:00 a.m. Rodent laboratory feed and drinking water were provided ad libitum. Young (3 month old) and aged (25 month old) pathogen-free Wistar rats were used throughout the experimentation. The body weight was monitored daily at 09:00 a.m. by weighing on a top-loading balance accurate to ± 0.1 g (Radwag, Poland). Only rats showing a regular and stabile 24-h body mass gaining were taken to the experiments.

All experimental procedures were approved by the Local Bioethical Committee for Animal Care (permission No 8/2011 and 7/2013).

### White blood cells (WBC) measurement

Blood samples were collected under general anesthesia with 3 % isoflurane either 48 h before the experiment or 4 h after LPS administration. The femoral vein was punctured with a 23-gage needle and blood was allowed to flow directly into tubes containing K_2_EDTA (ethylene diamine tetra acetic acid dipotassium salt). Analysis was performed within 10 min of blood collection using hematologic analyzer BC 2800 vet (Mindray, China).

### Liver glutathione assay

Liver samples (100 mg) were dissected out from anesthetized Wistar rats four hours after LPS injection. Next, they were rinsed twice with Phosphate-buffered saline and homogenized on ice in 1 mL of 5 % Sulfosalicylic Acid (Sigma-Aldrich, USA). Total glutathione contents were determined using Glutathione Assay Kit (Sigma-Aldrich, USA) according to the manufacturer’s protocol. Samples were assayed in triplicate. Colorimetric changes in the assays were detected using Synergy HT Multi-Mode Microplate Reader (BioTek Instruments, USA).

### Body temperature and motor activity measurement

Deep body temperature (Tb) and motor activity of the rats were measured using battery-operated telemetry transmitters (model TA-F40, Data Sciences International, USA) implanted intra-abdominally under sterile conditions. Before the implantation, the rats were anaesthetized with a mixture of ketamine (87 mg/kg) (Biowet, Poland) and xylazine (13 mg/kg) (ScanVet, Poland) injected intramuscularly. Then, following shaving and sterilization of a small abdomen surgical area, an incision was made in the skin and muscles of the abdomen, and a miniature temperature-sensitive telemetry device was placed into the peritoneal cavity. The muscle level of the abdomen and the skin were separately sutured closed. After surgery, rats were housed in individual plastic cages. The motor activity of the rats was detected by changes in the position of the implanted temperature-sensitive transmitter over the receiver board, resulting in a change of the signal strength that was detected by the external receiver antenna and recorded as a “pulse” or “count” of the activity. All experiments started at least 10 days after recovery from the surgical procedures.

### Induction of sickness behavior in the telemetry implanted rats

Lipopolysaccharide (LPS) extracted from *Escherichia coli* 0111:B4 (Sigma-Aldrich, USA) was dissolved in sterile 0.9 % sodium chloride (saline). Before injection, the stock solution of LPS (2.5 mg/ml) was warmed to 37 °C, diluted in a warm sterile saline to the desired concentration, and injected intraperitoneally (ip) at a dose of 50 μg/kg, as described previously (Wrotek et al. 2011, 2015). Rats were briefly restrained and not anesthetized during any injections. Immediately after the injections, rats were placed in their home cages.

### Interleukin (IL)-6 assay

Blood samples were collected via cardiac puncture onto the solution of ethylene diamine tetra acetic acid disodium salt (Na_2_EDTA, Sigma-Aldrich, USA) four hours post-injection of LPS from rats anesthetized with a mixture of ketamine/xylazine (87 mg/kg and 13 mg/kg, respectively, intramuscular injection). After centrifugation (20 min, 1500 g), the resulting plasma was stored at −20 °C until assay. The level of IL-6 was determined by a commercial ELISA kit (R&D System, Inc. USA), according to the manufacturer’s instructions. Colorimetric changes in the assay were detected using Synergy HT Multi-Mode Microplate Reader (BioTek Instruments, USA).

### Data analysis

Data are expressed as mean ± SE. Body temperature and motor activity data were recorded and computed at 5-min intervals using Data Acquisition Software (Data Sciences International, USA). For data analysis, excel plotting and presentation, the temperature recordings were pooled into 30-min averages, and the motor activity data were pooled into 12 h (day/night) averages.

A three way ANOVA (experimental groups × age × time of the day) wad applied to analyze significance of changes in body temperature and activity of rats. A two way ANOVA (experimental groups × age) was applied to analyze concentration of glutathione in the liver, IL-6 and WBC count in plasma. As a post hoc test the Tukey–Kramer (HSD) test was used. The threshold of statistical significance was p < 0.05 for all tests.

## Results

### White blood cells count (WBC) in old and young rats

Animals which WBC count was outside the norm (above 8.0 or under 2.0 cells [× 10^9^/L]) were excluded from our experiments. Analysis of variance showed that WBC was age dependent (F_(1.14)_ = 22.20, p < 0.001) and was affected by LPS administration (F_(1.14)_ = 13.43, p < 0.001), but there was no interaction between these two factors (F_(1.14)_ = 0.54, p < 0.006, n.s.).

**Fig. 1:**
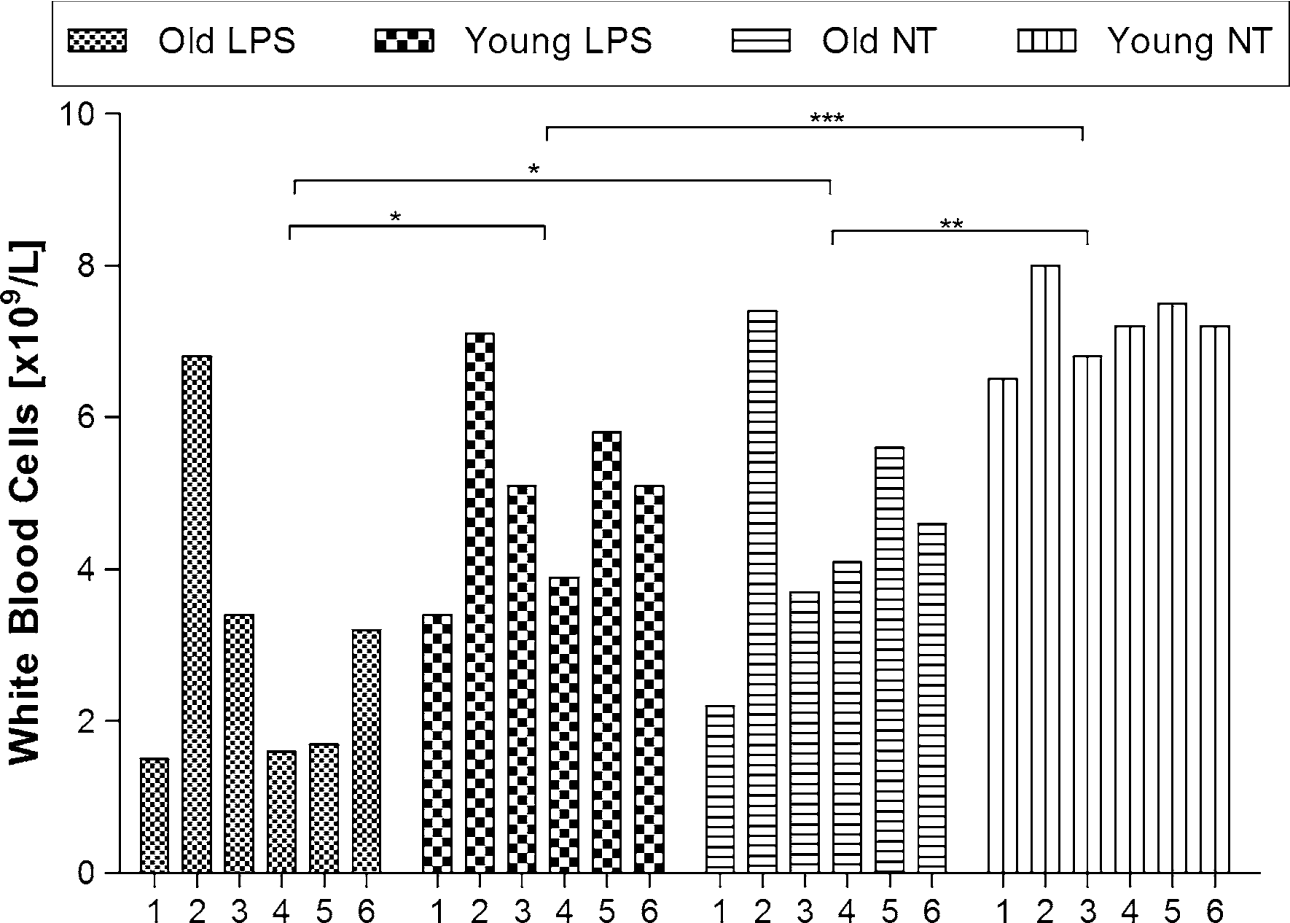
White blood cells count in old and young rats, treated with LPS or not-treated (NT). *Asterisks* indicates significant difference between the mean values calculated for groups of animals (* p < 0.05,**p < 0.01, ***p < 0.001). Numbers *1*–*6* indicate individual animal in a group

As can be seen on Fig. 1 in the old NT rats we noticed a reduced number of WBC compared to young NT animals (the mean value was 4.6 ± 0.70 × 10^9^/L vs 7.2 ± 0.21x10^9^/L, respectively, p < 0.001). After 4 h of LPS administration in both groups of animals a decrease in WBC was observed (to 2.9 ± 0.80 × 10^9^/L, p < 0.05, and to 5.1 ± 0.6 × 10^9^/L, p < 0.01, respectively).

### Changes of the rat liver glutathione contents in old and young rats

**Fig. 2:**
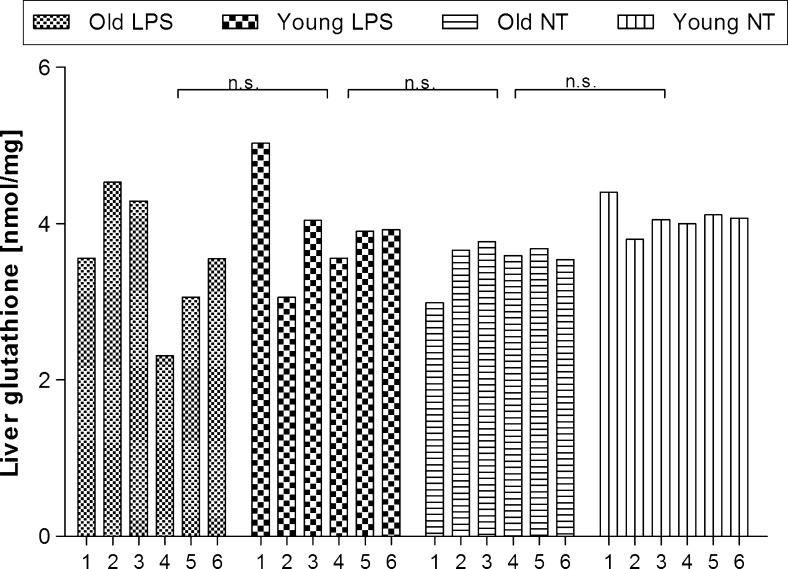
Liver glutathione concentration in old and young rats injected with endotoxin (LPS) or not-treated (NT). *n.s.* not significant. Numbers *1*–*6* indicate individual animal in a group

Figure 2 showed that in the healthy NT animals glutathione concentration did not differ significantly (F_(1.16)_ = 2.71, p < 0.11) between old and young rats (the mean value of glutathione concentration was 3.55 ± 0.14 nmol/mg in old NT rats and 4.07 ± 0.1 nmol/mg in young NT rats). Moreover, LPS administration did not affect significantly (F _(1.16)_ = 0.07, p < 0.79, n.s.) the liver glutathione level (the mean value of glutathione concentration was 3.60 ± 0.40 nmol/mg in old LPS-treated rats vs 3.50 ± 0.10 nmol/mg in old NT rats, and was 3.90 ± 0.33 nmol/mg in LPS-treated young rats vs 4.07 ± 0.10 nmol/mg in young NT rats). *Post*-*hoc* analysis showed also that mean values did not differ significantly.

### Body temperature (Tb) and motor activity of rats treated with LPS

Rats are nocturnal animals revealing low day-time and high night-time Tb. For our experiments we excluded rats that did not maintain those natural rhythm, and/or were apathetic (12 h mean value of motor activity was below 1.0 count during day-time and 2.5 counts during night-time).

Three way ANOVA showed that the Tb of selected rats was age-dependent (F_(1.568)_ = 53.43, p < 0.001), was affected by experimental conditions (F_(1.568)_ = 387.17, p < 0.001), and was also dependent on time of the day F_(1.568)_ = 171.13, p < 0.001).

Further analysis with HSD post hoc test showed that Tb during day,- and night-time in NT young rats were higher than that of recorded in old NT rats (12 h mean value of day-time Tb was 37.25 ± 0.03 °C in young rats vs 36.88 ± 0.03 °C in old rats and night-time Tb was 37.74 ± 0.03 °C vs 37.48 ± 0.040 °C, respectively, p < 0.001).

**Fig. 3:**
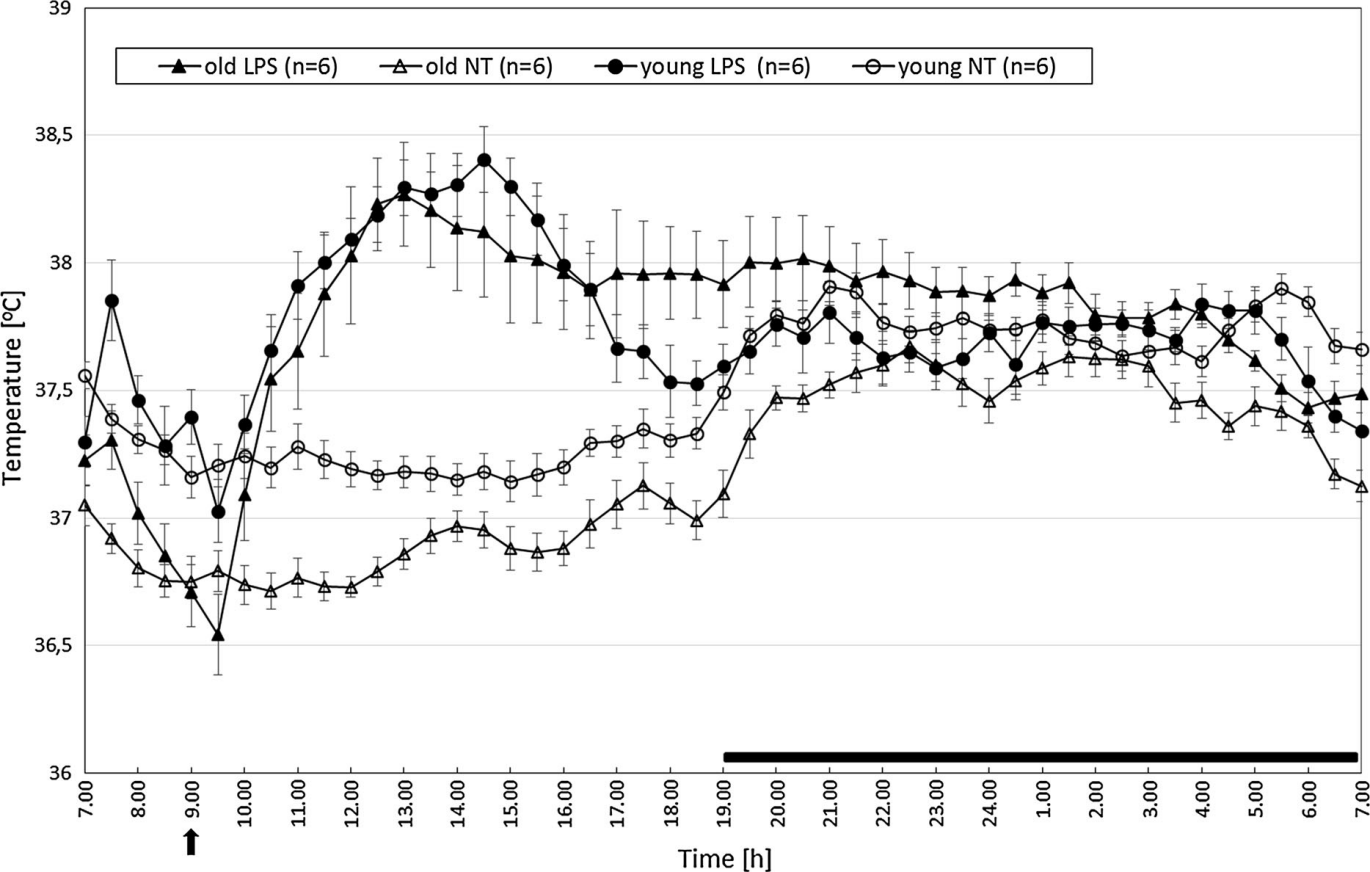
Changes of body temperature of old and young rats treated at 9:00 a.m. with LPS at a dose of 50 μg/kg (*arrowed*) versus not-treated rats (NT). Values are mean ± S.E. of 30-min averages. Letter *n* indicates sample size in a respective group. *Black horizontal line* shows lights-off period in a 12:12-h light dark cycle

In both young and old group of LPS-treated rats the day-time 12 h averages of Tb were significantly different (p < 0.001) from that recorded in NT rats (37.82 ± 0.15 °C in young LPS-treated rats vs 37.24 ± 0.01 °C in young NT rats, and 37.60 ± 0.26 °C in old LPS-treated rats vs 37.99 ± 0.04 °C in old NT rats). A standard LPS-induced fever in young rats was described previously (Wrotek et al. 2011, 2015). Intraperitoneal injection of the saline solution of LPS at a dose of 50 μg/kg induced fever in all rats (Fig. 3). In old LPS-treated rats fever did not reach the value observed in young rats (the mean value of Tb from 2nd to 6th hour after LPS injection was 38.06 ± 0.01 °C in old rats, whereas in young LPS-treated rats it was 38.19 ± 0.06 °C, p < 0.01).

Then, there was a gradual decrease of Tb towards its normal value. During night-time Tb of old LPS-treated rats was still higher than that in NT old rats and LPS-treated young rats (12 h mean value of night-time Tb was 37.82 ± 0.09 °C for the old LPS-treated animals, 37.47 ± 0.04 °C for old NT animals was, and 37.69 ± 0.04 °C for the young LPS-treated animals, p < 0.001).

Three way ANOVA showed that activity was age dependent (F_(1.572)_ = 76.21, p < 0.001), was affected by experimental conditions (F_(1.572)_ = 17.39, p < 0.001), and was also dependent on time of the day (F_(1.572)_ = 221.56, p < 0.001), but there was no interaction between these factors (F_(1.572)_ = 1.41, p < 0.23, n.s.). *Post*-*hoc* analysis showed that in all experimental groups (apart from old LPS-treated rats), night-time motor activity was higher (p < 0.001) than that recorded in day-time.

**Fig. 4:**
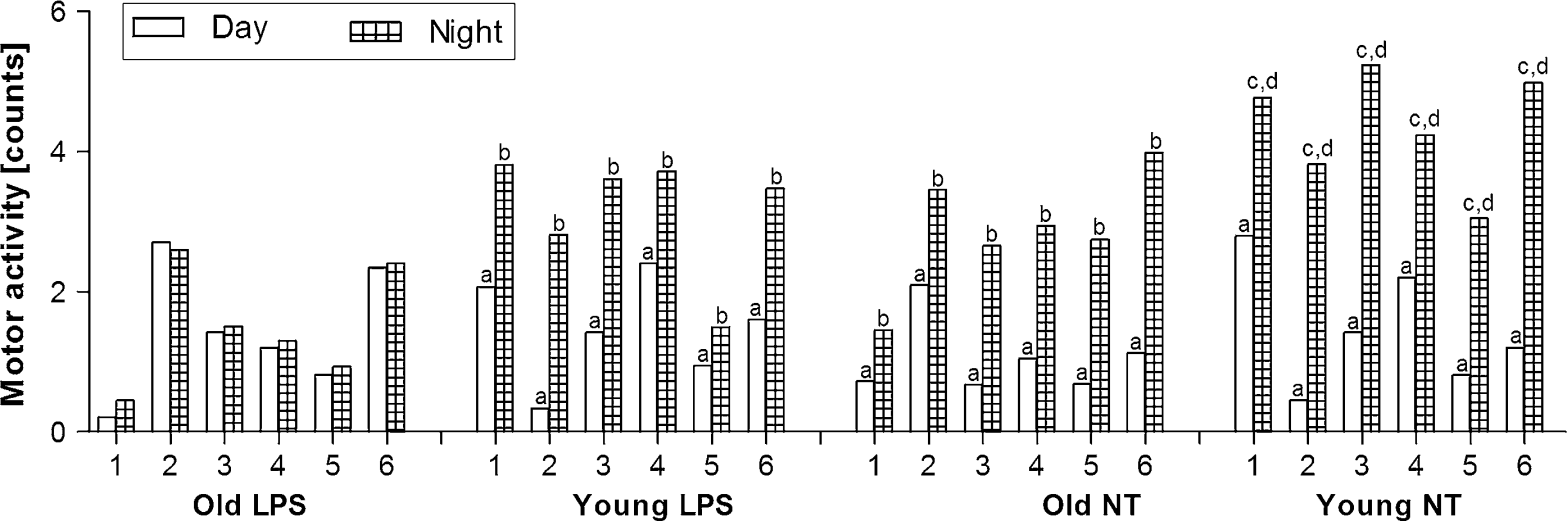
12-h averages of day-time and night-time motor activity of rats treated with LPS, or not-treated (NT). Numbers *1*–*6* indicate individual animal in a group. *Letters* indicate significant difference: **a** significant differences between day-time and night-time motor activity in each animal in a group (p < 0.001); **b** significant differences in night-time motor activity between young LPS, old NT compared with old LPS rats (p < 0.001); **c** significant differences in night-time motor activity between young NT compared with young LPS-treated rats (p < 0.01) and **d** significant differences in motor activity between young NT and old NT rats (p < 0.001)

Activity data (Fig. 4) followed the temperature changes, with low day-time values (12 h day-time mean values were: 1.05 ± 0.29 counts in old NT rats, and 1.47 ± 0.49 counts in young NT animals), and high night-time values (12 h night-time mean values were 2.74 ± 0.53 counts and 4.29 ± 0.52, respectively). Post-hoc analysis showed that LPS administration did not affect significantly day-time motor activity (12 h mean values of day-time activity were 1.45 ± 0.62 counts in old rats and 1.51 ± 0.69 counts in young rats versus 1.05 ± 0.29 counts and 1.47 ± 0.49 counts for their NT counterparts, p < 0.32, n.s.), but night-time activity in both LPS-treated groups were decreased in compare to their NT counterparts (12 h mean values were 1.56 ± 0.40 counts vs 2.74 ± 0.53 counts for old rats and 3.44 ± 0.60 counts vs 4.28 ± 0.57 counts for young rats, p < 0.001).

### Effect of LPS on IL-6 level in old and young rats

**Fig. 5:**
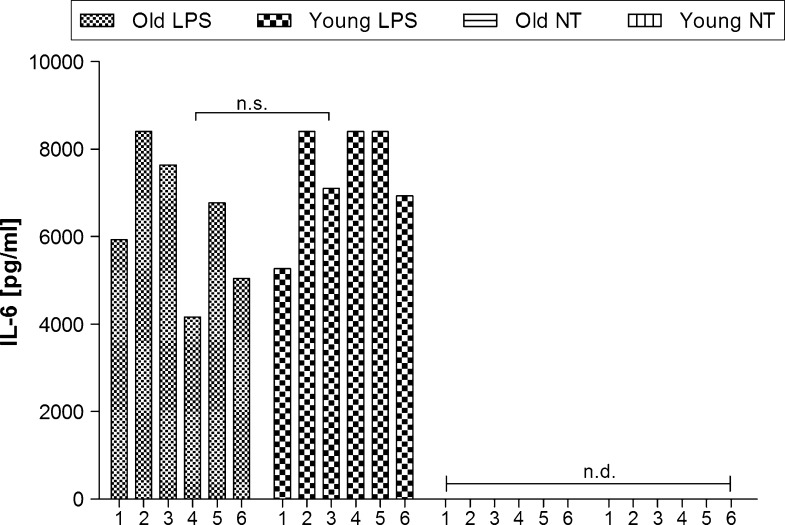
The effect of LPS on plasma IL-6 level. Asterisks indicate significant difference between the mean values for respective groups. *1*–*6* indicates individual animal in a group. *n.d.* not detected

The basal level of plasma IL-6 was below the lowest detectable standard. The analysis of variance showed that injection of LPS provoked an elevation of IL-6 level in both groups of animals (F_(1.24)_ = 380.29, p < 0.001) to 6322.82 ± 537.00 pg/mL in old LPS-treated and 7415.62 ± 451.88 pg/mL in young LPS-treated rats (Fig. 5).

## Discussion

In the present study, we demonstrated that physiological rhythm of body temperature and motor activity are decreased in old male rats (Figs. 3, 4, respectively). On the other hand, the liver glutathione concentration did not differ significantly between old and young rats (Fig. 2). Old rats were able to develop endotoxic fever, although its pattern changed compared to the endotoxic fever observed in young rats. It means that body temperature during fever in old rats was significantly lower than that recorded in young rats. Furthermore, we showed that LPS injection provokes a significant increase in plasma IL-6 in both old and young animals (Fig. 5).

The most common hypothesis of aging is based on age-related alterations in cellular redox balance (Sohal and Orr 2012) which are accompanied by age-related dysregulation of the immune system (Chung et al. 2009). In consequence, advanced age is known as a critical risk factor for developing health complications, including infections and delirium that often result in further comorbidities and higher mortality rates (Gavazzi et al. 2004; Inouye et al. 2014). In organisms that suffer from various diseases weakened antioxidative protection is often observed (Townsend et al. 2003). Similarly, it is commonly accepted that glutathione, the major intracellular redox buffer, is decreased in aged organisms (Liu and Choi 2000, Liu et al. 2004, Mosoni et al. 2004). Whether lowered glutathione is the primary cause, or is a downstream consequence of the pathological processes is still under debate. Therefore, in our experiments we made an effort to eliminate rats which had an uncertain status of health. We observed that liver glutathione content in our healthy old rats is similar to that in young rats. It must be emphasized that our observation is consistent with results shown by Nakata et al. (1996) who also did not observe any statistically significant change in rodents during aging to 30 months. Our data suggest that advanced age is not necessarily associated with low glutathione level. We suppose that inconsistent results concerning glutathione concentrations in old rats, which were provided by various authors, may by a consequence of subclinical disease which was not taken into account by researchers.

It is well known that body temperature of older men and women is lower than that in younger people (Lu et al. 2010). Moreover, the circadian rhythm of body temperature is thought to be generated by an endogenous component and depends on motor activity (Refinetti 1994; Weinert and Waterhouse 1998). In our research we observed that old rats are less active and in fact have lower basal body temperature in comparison to young animals. We suppose that different body weight and/or body compositions may be responsible for this effect, especially since it is known that the percent mass of skeletal muscle declines in old age (Delp et al. 1998).

Thermoregulatory behavior plays a significant role in fever production. Therefore, one may expect that fever in old rats, whose basal body temperature differs from that observed in young rats, changes. Indeed, in a current paper, we present that healthy aged rats develop reduced fever, furthermore its pattern is different from that in young rats. These results are consistent with data published by others (Florez-Duquet et al. 2001; Tocco-Bradley et al. 1985; Foster et al. 1992), however those authors presented data recorded for only 7 h after injecting LPS, thus they were unable to observe full course of fever. Moreover, changes in motor activity were not analysed at all.

In our previous paper (Wrotek et al. 2015) we have shown that pharmacological decrease of glutathione level in young rats resulted in attenuated fever. Therefore, we supposed that glutathione is strongly associated with fever. Surprisingly, in the current experiments we have found that fever in old rats was attenuated despite unchanged glutathione level. We conclude, therefore, that fever in old rats is reduced independently of glutathione.

IL-6 plays an important role as an endogenous mediator in LPS-induced fever. The presence of IL-6 is critical for febrile response, as seen by the absence of the endotoxic fever in IL-6 knock-out mice (Chai et al. 1996, Kozak et al. 1998). In our investigation we observed that the plasma level of IL-6 in old LPS-treated rats is similar to that observed in young LPS-treated rats. These data suggest that IL-6 is not responsible for the changed pattern of endotoxic fever in old rats.

The accumulated data suggest that fever has a protective role in promoting host defence against infection (Kluger et al. 1996; El-Radhi 2012). It is likely that aged rats had been exposed to brief periods of stress many times in their life, so that their stress response-induced gene expression was stimulated and the related pathways of maintenance and repair were enhanced. Such a phenomenon in which stimulatory responses to low doses of otherwise harmful conditions have beneficial biological effects is known as hormesis (Rattan 2001; Calabrese et al. 2015). Although in our experiments we did not analyse the data obtained on rats with ongoing inflammation, we cannot exclude that rats used in these experiments during their long lifespan were stimulated many times by various stressors such as pathogens and temperature changes. In accordance with theory of hormesis, we suppose that such mild, repeated stresses are responsible for adaptation of rats to subsequent stresses, e.g. lipopolysaccharide challenge. In consequence, although old rats tested in our experiments have shown lower motor activity and basal Tb comparing with young rats, they were still able to develop SB.

The current experiments allow to conclude, that pyrogenic dose of endotoxin provokes SB which is different in old and young rats. Furthermore, in old rats changed pattern of fever is not exactly associated with glutathione level. Because the reason of differences between fever in both old and young rats is still unknown, further research is needed.
